# Vaginal Dysbiosis and Partial Bacterial Vaginosis: The Interpretation of the “Grey Zones” of Clinical Practice

**DOI:** 10.3390/diagnostics11020191

**Published:** 2021-01-28

**Authors:** Giuseppina Campisciano, Nunzia Zanotta, Vincenzo Petix, Manuela Giangreco, Giuseppe Ricci, Gianpaolo Maso, Manola Comar, Francesco De Seta

**Affiliations:** 1 Advanced Laboratory of Translational Microbiology, Institute for Maternal and Child Health—IRCCS “Burlo Garofolo”—Via dell’Istria 65, 34137 Trieste, Italy; giusi.campisciano@burlo.trieste.it (G.C.); nunzia.zanotta@burlo.trieste.it (N.Z.); vincenzo.petix@burlo.trieste.it (V.P.); 2 Institute for Maternal and Child Health—IRCCS “Burlo Garofolo”—Via dell’Istria 65/1, 34137 Trieste, Italy; manuela.giangreco@burlo.trieste.it; 3 Obstetrics and Gynecology, Institute for Maternal and Child Health—IRCCS “Burlo Garofolo”—Via dell’Istria 65, 34137 Trieste, Italy; giuseppe.ricci@burlo.trieste.it (G.R.); gianpaolo.maso@burlo.trieste.it (G.M.); francesco.deseta@burlo.trieste.it (F.D.S.); 4 Department of Medical, Surgical and Health Sciences, University of Trieste, Strada di Fiume, 447, 34149 Trieste, Italy

**Keywords:** bacterial vaginosis, aerobic vaginitis, STIs, Nugent score, intermediate vaginal microbiota

## Abstract

Bacterial vaginosis (BV) affects one-third of reproductive age women, increasing the risk of acquiring sexually transmitted infections (STIs) and posing a risk for reproductive health. The current diagnosis with Gram stain (Nugent Score) identifies a transitional stage named partial BV or intermediate microbiota, raising the problem of how to clinically handle it. We retrospectively analyzed cervicovaginal swabs from 985 immunocompetent non-pregnant symptomaticspp. women (vaginal discharge, burning, itching) by Nugent score and qPCR for BV, aerobic or fungal vaginitis, and STIs (*Mycoplasmas* spp., *Chlamydia t.*, *Trichomonas v.*, and *Neisseria g.*). Nugent scores 0–3 and 7–10 were confirmed in 99.3% and 89.7% cases, respectively, by qPCR. Among Nugent scores 4–6 (partial BV), qPCR identified 46.1% of BV cases, with 37.3% of cases negative for BV, and only 16.7% of partial BV. Gram staining and qPCR were discordant (*p* value = 0.0001) mainly in the partial BV. Among the qPCR BV cases, the presence of aerobic vaginitis and STIs was identified, with a significant association (*p* < 0.0001) between the STIs and partial BV/overt BV. qPCR is more informative and accurate, and its use as an alternative or in combination with Gram staining could help clinicians in having an overview of the complex vaginal microbiota and in the interpretation of partial BV that can correspond to vaginitis and/or STIs.

## 1. Introduction

Bacterial vaginosis (BV) is a polymicrobial syndrome characterized by a reduction of vaginal lactobacilli, elevated pH and a concomitant overgrowth of strict or facultative anaerobic bacteria, such as *Gardnerella vaginalis* [1]. In recent years, BV has been associated with several clinical complications such as preterm birth, pelvic inflammatory disease, cervicitis, and cervical intraepithelial neoplasia [2,3]. Moreover, BV increases the risk of acquisition of sexually transmitted infections (STIs), including human immunodeficiency virus (HIV), papillomavirus, *Chlamydia trachomatis* and *Neisseria gonorrhoeae* [4,5,6,7].

Risk factors for BV are multiple sexual partners, smoking, and decreased *Lactobacillus* species with concurrent colonization with *Candida* species [8]. Similar risk factors predispose to aerobic vaginitis (AV), which is a type of vaginal dysbiosis often overlooked and that can be misdiagnosed as BV. AV is often characterized by more extreme inflammatory changes than BV and by the presence of mainly aerobic enteric commensals or pathobionts, including Group B *Streptococcus* (*S. agalactiae*), *Enterococcus faecalis*, *Escherichia coli*, and *Staphylococcus aureus* [9], living as non-harming symbionts under normal circumstances [10]. These pathobionts occur at lower levels than BV-anaerobes but with a higher pathogenic potential. Because pathobionts rarely dominate, their presence is often overlooked despite the fact that these lower levels may be clinically relevant [11].

BV is diagnosed by clinical (Amsel criteria) or microscopic findings (Hay’s criteria or Nugent score). The Nugent scoring is a Gram stain scoring system of vaginal smears and, currently, the gold standard laboratory method that detects the state of vaginal health based on the visualization and quantification of the numbers of Gram-positive morphotypes (*Lactobacillus* spp.) respect to the number of Gram-negative or Gram-variable morphotypes (*Gardnerella* and *Mobiluncus*) [12].

In recent years, with the advent of molecular techniques, it has been observed that several novel bacteria, including *Atopobium vaginae* and *Prevotella bivia*, are associated with BV [13]. In keeping with this, the use of the Nugent score is limited as it does not permit the identification of many bacterial species associated with BV. Moreover, the percentage of samples to which the intermediate vaginal microbiota category is assigned by Nugent score may exceed 20%, and it remains debated how to handle these results [14]. Since BV may have an adverse effect on women’s reproductive health, it is necessary to use rapid, accurate, and objective methods for diagnosis.

Recently, new methodologies based on molecular techniques, such as qPCR, have been introduced to detect and quantify the presence of most common microorganisms associated with BV and with AV. These assays are fast and have shown high sensitivity and specificity. Moreover, molecular techniques help to uncover pathogens not identifiable with current diagnostic methods, to determine the number of microorganisms and assess the ratio between the different groups of conditionally pathogenic microorganisms and the normal microbiota [15,16,17]. These new molecular techniques can better clarify the qualitative and quantitative variations of the vaginal microbiota, helping clinicians in the interpretation of some “grey zones” of clinical practices such as intermediate Nugent scores or dysbiosis.

The present study aimed to compare the Nugent scoring method for the diagnosis of BV with the quantitative bacterial species/genus-specific real-time PCR assays (qPCR), by investigating the microbiological composition of cervicovaginal swabs from women of reproductive age. The Nugent score diagnosis has been coupled with comprehensive qPCR panels of pathogens associated with BV, AV, candidiasis, and STIs. In addition, the association between the presence of BV and the co-presence with STIs, candidiasis, and vaginitis has been surveyed.

## 2. Materials and Methods

### 2.1. Patients and Samples

Our retrospective study included a total of 985 immunocompetent symptomatic low-risk women. All women were reported to have clinical symptoms (vaginal discharge, burning, itching) and they attended the Infection Gynecology Service of the Institute for Mother and Child Health IRCCS Burlo Garofolo of Trieste, Italy, as outpatients. The study was approved by the local ethics board and written informed consent was obtained from all subjects.

The inclusion criteria were Caucasian ethnicity, reproductive age, at least 18 years old, not pregnant, no current use of hormonal or barrier contraceptive products, vaginal douching, tobacco or alcohol abuse, no hospitalization or systemic use of medication for chronic diseases or antibiotics/probiotics within the 6 months before sample collection, and no intercourse in the day before sampling.

Vaginal samples were collected using a 200 mm polyethylene Cervex brush device (Rovers Medical Devices B.V., Oss, North Brabant, The Netherlands) by a single gentle 360° rotation of the cytobrush at the vaginal wall, under speculum examination. Two vaginal swabs were performed on the lateral vaginal wall, one for qPCR tests and the second one for Nugent score. Cervicovaginal cells were obtained reaching the endocervical canal and touching both the ectocervical area and the transformation zone (T-zone). Swabs were suspended in 1.5. mL of TE buffer and stored at −80 °C.

### 2.2. Detection of STIs and Diagnosis of BV, Vaginitis, and Candidiasis

DNA was extracted from cervical and vaginal samples using the Maxwell CSC Blood DNA Kit for the Maxwell CSC Instrument (Promega, Madison, WI, USA) as indicated by the supplier, and stored at −80°C. The presence of *C. trachomatis*, *Mycoplasma* (*hominis/genitalium*), *Ureaplasma* (*parvum/urealyticum*), *Neisseria gonorrhoeae*, and *Trichomonas vaginalis* DNA was detected by Real Time PCR (RT-PCR) using a commercial kit (RealLine Pathogen Diagnostic Kits, Bioron Diagnostics).

For the diagnosis of BV, the commercial qPCR kit Bacterial Vaginosis Real-TM Quant was used, detecting *Gardnerella vaginalis*, *Atopobium vaginae*, *Lactobacillus* spp., and the total bacterial load (Sacace Biotechnologies, Como, Lombardia, Italy). According to this test, samples can result as positive/negative for BV or positive for partial BV (defined as a decrease of lactobacilli and an increase of *Atopobium* and *Gardnerella* that does not exceed the amount of lactobacilli). In parallel, the Nugent score on Gram-stained vaginal smears was calculated by assessing the numbers of *Lactobacillus* morphotypes (scored as 0 to 4), *G. vaginalis* morphotypes (scored as 0 to 4), and *Mobiluncus* morphotypes (scored as 0 to 2). A score of 0–3 was categorized as normal microbiota, 4–6 as intermediate microbiota, and 7–10 as BV. If the quality of the slide was poor, the slide was classified as indeterminate.

For the diagnosis of AV, the commercial qPCR kit ProstateScreen Real-TM was used, detecting Escherichia coli, Enterobacter/Klebsiella, Proteus spp., Serratia spp., Pseudomonas aeruginosa, Enterococcus faecium/faecalis, Staphylococcus aureus, and Streptococcus spp. (Sacace Biotechnologies, Como, Lombardia, Italy) whereas, for the diagnosis of candidiasis, the detected species using the commercial qPCR kit Candidosis Real-TM Quant were Candida albicans, Candida glabrata, Candida krusei, and Candida parapsilosis tropicalis (Sacace Biotechnologies, Como, Lombardia, Italy).

All the amplification and PCR product detection was performed by the CFX96™ instrument (Bio-Rad, Hercules, CA, USA).

### 2.3. Statistical Analysis

Frequencies and percentages to describe the distribution of categorical variables (e.g., yes/no) and median and interquartile range for continuous variables (e.g., bacterial load) were calculated. To evaluate significant differences in our cohort between categorical variables, Chi-square or exact Fisher tests were applied. The Wilcoxon–Mann–Whitney non-parametric test was used to identify the difference between groups in the distribution of a continuous variable. To establish if the Gram test agreed with the molecular test in discriminating vaginosis from non-vaginosis patients or intermediate situations, Cohen’s Kappa coefficient was calculated. A *p*-value < 0.05 was considered as statistically significant. Statistical analysis was performed using SAS software, Version 9.4 (SAS Institute Inc., Cary, NC, USA).

## 3. Results

Out of the 985 women included in the present study, 156 samples were not tested for AV, 196 samples were not tested for *Candida* spp., 1 sample was not tested for STIs (*Chlamydia t.*, *Neisseria g.*, *Trichomonas v.*, *Mycoplasmas/Ureaplasmas*).

Based on the PCR molecular test diagnosis, out of the 985 women tested for the BV qPCR panel, 723 (73.4%) women were negative for BV, 214 (21.7%) were positive for BV, and 48 (4.9%) showed a partial BV (defined as a decrease of lactobacilli and an increase of *Atopobium* and *Gardnerella* that does not exceed the amount of lactobacilli). Among the 723 women negative for BV, 296 (40.9%) had aerobic vaginitis, 93 (12.8%) had candidiasis, and 351 (48.5%) tested positive for one or more STIs, including *Chlamydia t.*, *Neisseria g.*, and *Ureaplasmas/Mycoplasmas*. In some cases, women tested positive for more than one qPCR panel.

Concerning the 214 women diagnosed with BV, 84 (39.2%) had a concomitant vaginitis/coinfection, 27 (12.6%) had candidiasis, and 206 (96.2%) tested positive for STIs, mainly *Mycoplasmas* spp., and specifically, *M. hominis* (*n* = 36), *M. genitalium* (*n* = 3), *U. urealyticum* (*n* = 24), and *U. parvum* (*n* = 143). In some cases, women tested positive for more than one qPCR panel. In this group, only few coinfections with *Trichomonas vaginalis* (*n* = 10), *Chlamydia trachomatis* (*n* = 16), and *Neisseria gonorrhoeae* (*n* = 4) were identified.

Among the women with a partial BV (*n* = 48), 17 (35.4%) had aerobic vaginitis, 10 (20.8%) had candidiasis, and 45 (93.6%) tested positive for STIs. More precisely, 18 women tested positive for *U. parvum*, 1 for *U. urealyticum*, and 3 for *T. vaginalis* while there were no positive samples for *C. trachomatis*, *N. gonorrhea*, and *Mycoplasmas*. Several of the tested microorganisms were co-detected. To note, a significant association (*p* < 0.0001) between the positivity for STIs and partial BV/overt BV has been observed (Table 1).

A total of 611 samples were tested in parallel with the qPCR method and with the Gram staining test for the diagnosis of BV. According to the Cohen’s kappa coefficient (κ) test, the qPCR assay confirmed the diagnosis of 399 (99.3%) women negative for BV with Nugent score 0–3 and 96 (89.7%) women with BV and Nugent score 7–10. In the intermediate results, defined by the Gram staining with Nugent score 4–6, the qPCR assay identified 47 (46.1%) women affected with BV, mainly due to *Atopobium*, 38 (37.3%) negative for BV, and 17 (16.7%) with a partial BV. Overall, the qPCR test and the Gram staining test were slightly discordant (*p* value = 0.0001) but the major discordance was observed in the intermediate clinical picture (Table 2).

An association between the bacterial load determined by the qPCR test on the vaginal swab and the diagnosis of coinfections (aerobic vaginitis and candidiasis) has been observed. More precisely, among the women positive for BV (*n* = 214), a higher bacterial load was associated with the presence of aerobic vaginitis (*p* = 0.0002) and candidiasis (*p* = 0.0014). The bacterial load did not significantly differ about the positivity for STIs (Table 3).

The possible association between a specific *Candida* species and the presence of BV has been tested but no significant association has been found (Table 4). The same test has been performed for the bacterial species responsible for vaginitis and no significant association was identified (Table 5).

## 4. Discussion

Bacterial vaginosis is the most common lower genital tract infection among women of reproductive age, increasing the risk for the acquisition of STIs and, in turn, posing a risk for reproductive outcomes by promoting the bacterial colonization of the upper reproductive tract [18,19]. BV is usually diagnosed by performing a Gram staining of vaginal smears, with a sensitivity ranging from 62% to 100% and a positive predictive value ranging from 76% to 100% [20]. Although Gram staining is a simple and fast procedure, it requires a trained microbiologist to be accurate and for the interpretation of results to be consistent.

According to Gram staining, the diagnosis can be divided into three groups: normal vaginal microbiota, intermediate microbiota, and bacterial vaginosis. Treatments are advised for women with symptoms or with a positive test for BV. Patients in the intermediate categories are generally not considered for treatment [21] and are the object of clinical debate on whether to consider them as having a physiologic or pathologic status.

In our study, we observed a misdiagnosis of the intermediate microbiota by the Gram staining, which was not confirmed by the qPCR method. Out of the 102 women diagnosed as intermediate by Gram staining, only 17 (16.7%) were confirmed by qPCR as an intermediate clinical picture (partial BV) while 38 (37.3%) were negative for BV and 47 (46.1%) were affected with BV. The women negative for BV diagnosed based on the qPCR showed the presence of *Candida* spp. (*n* = 12) while the women diagnosed with BV showed the co-presence of aerobic vaginitis (AV) (*n* = 26) with species as *Escherichia*, *Enterococcus*, *Staphylococcus*, and *Streptococcus*, in addition to STIs such as *Trichomonas* and *Ureaplasmas*. Thus, the qPCR suggested an alternative diagnosis, due to being able to identify targets which are ambiguous or impossible to discern by Gram staining: the presence of *Atopobium* and/or coinfections. Generally, women with intermediate Nugent scores are not usually recommended conventional antibiotics treatments [22], unless patients are symptomatic or are young women with a high risk of acquisition of STIs.

Sometimes, in these situations, some of the symptomatic relapses are due to the worsening of a preexisting underdiagnosed dysbiotic state rather than a relapsing of a new episode of BV.

Another consideration is the high prevalence of AV pathogens among the intermediate Nugent score (Table 1). This result suggests that AV is underreported and underdiagnosed at present. This point is clinically relevant as BV treatments are not effective for AV [23,24]. Thus, including AV in the diagnostic workup for patients showing vaginitis symptoms would be highly recommendable. As no significant association between AV and BV has been detected, this result highlights that the presence of AV is an independent factor from BV. The increased bacterial load is suggestive for the co-presence of AV and BV, as shown in Table 3. In our cohort, we observed that, in the case of BV, a higher probability of co-detection of *Candida* and AV exists when the total bacterial load is high. The quantification of the bacterial load is achieved using the qPCR, providing additional information that is not obtainable from the Gram staining. While taking into consideration that the total bacterial load may not consistently improve diagnostic accuracy, it could help the predictive values as it has been seen that elevated loads of *G. vaginalis* and *A. vaginae* are predictive of bacterial vaginosis [25].

In general, the status of vaginal microbiota is normal or dysbiotic depending on the ratio between lactobacilli and dysbiosis-associated anaerobes/aerobes and/or on the co-presence of other microorganisms as Candida or STIs. A decrease in lactobacilli is detected both by Gram staining and by qPCR but with the latter technique being more sensitive. qPCR can detect adjunctive bacteria, such as *Atopobium* spp., and gives the best results especially when aerobes, alone or in combination with anaerobes, are detectable in the vaginal swab, such as the cases of AV. On the other hand, we know that BV is a polymicrobial syndrome. Thus, the multiplex qPCR, targeting a limited number of microorganisms, can identify a partial BV or non-specific dysbiosis. For this reason, it should be coupled with other multiplex molecular panels such as those for the detection of *Candida* spp., AV pathogens, and STIs. This is the only way to obtain a clearer overview of cervicovaginal microbiota in symptomatic cases and to better clinically define the patients with this problem.

Indeed, the partial BV (defined as a decrease of lactobacilli and an increase of *Atopobium* and *Gardnerella* that does not exceed the amount of lactobacilli) or overt BV significantly predispose to the acquisition of exogenous infections, as seen in our cohort, where a significant association (*p* < 0.0001) between the positivity for STIs and partial BV/overt BV has been observed. To note, the association between STIs and partial BV/overt BV is justified by the presence of *U. parvum* (*n* = 18), *U. urealyticum* (*n* = 1) and *T. vaginalis* (*n* = 1) among patients. Especially concerning *Ureaplasmas*, although many studies have addressed their possible role in the vaginal dysbiosis, many aspects remain to be elucidated [26].

## 5. Conclusions

In conclusion, our clinical practice with Gram staining could benefit from more advanced and accurate molecular tests, which can simultaneously detect a plethora of microorganisms. This is particularly important in the intermediate Nugent score which can hide some other vaginitis such as candidiasis, AV, or STIs. Nowadays, several qPCR panels, with improved accuracy, have been developed to target multiple microorganisms associated with vaginal dysbiosis. Although Gram staining has a lower cost than any molecular test, the interpretation of vaginal smears is biased by subjectivity. Conversely, molecular tests bypass this problem and can be very useful for their predictive values of relapse of BV, being able to detect very low amounts and co-infections. In addition, a great advantage is the possibility to obtain from vaginal swab both quantitative and qualitative information on vaginal microbiota and, in case of partial BV, it is reasonable to search for AV pathogens and *Candida* spp. and to test a cervical swab for STIs. Therefore, the possibility to test women simultaneously for BV and AV pathogens and STIs could be helpful to easily screen cervicovaginal swabs of high-risk patients, of sexually active women below 25 years, and in all recurrent cervicovaginal infections. The main drawback for the development of new diagnostic molecular panels for BV is the lack of a uniform case definition and that the etiology of BV remains poorly understood [27,28]. For this reason, continuous progress is needed to understand the complexity of BV, especially in those cases in which an intermediate score has been assigned and molecular tests do not identify a specific pathogen.

## Figures and Tables

**Table 1:** Association between the diagnosis of bacterial vaginosis, based on qPCR test, and the presence of co-infections.

		Bacterial Vaginosis			Total		Chi square or * Fisher Test
		Negative	Partial	Positive			
		n (%)					
**^1^ AV**	**NO**	319 (51.9)	19 (52.8)	94 (52.8)	432		0.97
	**YES**	296 (48.1)	17 (47.2)	84 (47.2)	397		
	**Not included**	108	12	36			
**^2^*Candida* spp.**	**NO**	482 (83.8)	31 (75.6)	146 (84.4)	659		0.37
	**YES**	93 (16.2)	10 (24.4)	27 (15.6)	130		
	**Not included**	148	7	41			
**^3^ STIs**	**NO**	372 (51.5)	1 (2.2)	8 (3.7)	382		<0.0001 *
	**YES**	351 (48.5)	46 (97.8)	206 (96.3)	602		
	**Not included**		1				

^1^ 156 samples not tested for AV, ^2^ 196 samples not tested for *Candida* spp., ^3^ 1 sample not tested for STIs (*Chlamydia t.*, *Neisseria g.*, *Trichomonas v.*, *Mycoplasmas/Ureaplasmas*).

**Table 2:** Agreement between Nugent scoring and qPCR test results. The Cohen’s kappa coefficient (κ) was used to test the agreement between two different diagnostic methods for bacterial vaginosis.

Nugent Scoring—n (%)	BV qPCR—n (%)			
	Positive	Partial BV	Negative	Total
Positive (7–10)	96 (89.7)	7 (6.5)	4 (3.7)	107
Intermediate (4–6)	47 (46.1)	17 (16.7)	38 (37.3)	102
Negative (0–3)	3 (0.8)	0 (0.0)	399 (99.3)	402
**Total**	146	24	441	* 611
	κ = −0.0937, *p* value < 0.0001			

* 114 samples were excluded from the comparison as they were not classifiable according to the Gram staining.

**Table 3:** Association between bacterial load determined by the qPCR test and the diagnosis of AV and candidiasis.

Co-Infections		Total Bacterial Load Median Value (25th–75th Quartile)	Wilcoxon–Mann–Whitney Test
AV	NO	10^8 (7–8)^	0.0002
	YES	10^8 (8–9)^	
*Candida* spp.	NO	10^8 (7–9)^	0.0014
	YES	10^9 (8–9)^	
STIs	NO	10^8 (7–8)^	0.44
	YES	10^8 (7–9)^	

**Table 4:** Association between a specific *Candida* species and the presence of bacterial vaginosis.

*Candida* spp.		BV qPCR Results–n (%)			Total	Chi Square or * Fisher Test
		Negative	Partial BV	Positive		
^1^ *C. albicans*	NO	482 (84)	33 (78.6)	146 (85.4)	661	0.56
	YES	92 (16)	9 (21.4)	25 (14.6)	126	
^2^ *C. glabrata*	NO	561 (97.7)	42 (100)	171 (99.4)	774	0.38 *
	YES	13 (2.3)	0 (0.0)	1 (0.6)	14	
^3^ *C. krusei*	NO	572 (99.8)	42 (100)	171 (99.4)	785	0.47 *
	YES	1 (0.2)	0 (0.0)	1 (0.6)	2	
^4^*C. parapsilosis*, *C. tropicalis*	NO	573 (99.6)	42 (100)	172 (100.0)	787	1.00 *
	YES	2 (0.4)	0 (0.0)	0 (0.0)	2	

^1^ 198 missing samples, ^2^ 197 missing samples, ^3^ 198 missing samples, ^4^ 196 missing samples.

**Table 5:** Association between a specific species responsible for vaginitis and the presence of bacterial vaginosis.

Microorganism		BV qPCR Results–n (%)			Total	Chi Square or * Fisher Test
		Negative	Partial BV	Positive		
^1^ *E. coli*	NO	533 (87.4)	31 (86.1)	162 (91.5)	726	0.28 *
	YES	77 (12.6)	5 (13.9)	15 (8.5)	97	
^2^ *Enterobacter/Klebsiella*	NO	596 (97.2)	36 (100.0)	173 (98.3)	805	0.61 *
	YES	17 (2.8)	0 (0.0)	3 (1.7)	20	
^3^*Proteus* spp.	NO	613 (100.0)	36 (100.0)	176 (100.0)	825	NA
	YES	0 (0.0)	0 (0.0)	0 (0.0)	0	
^4^*Serratia* spp.	NO	613 (100.0)	36 (100.0)	176 (100.0)	825	NA
	YES	0 (0.0)	0 (0.0)	0 (0.0)	0	
^5^ *P. aeruginosa*	NO	613 (100.0)	36 (100.0)	175 (100.00)	824	NA
	YES	0 (0.0)	0 (0.0)	0 (0.0)	0	
^6^ *E. faecium/faecalis*	NO	584 (95.3)	35 (97.2)	167 (94.9)	786	0.95 *
	YES	29 (4.7)	1 (2.8)	9 (5.1)	39	
^7^ *S. aureus*	NO	607 (99.0)	36 (100.0)	174 (98.9)	817	1.00 *
	YES	6 (1.0)	0 (0.0)	2 (1.1)	8	
^8^*Streptococcus* spp.	NO	346 (56.4)	18 (50.0)	101 (57.4)	465	0.72
	YES	267 (43.6)	18 (50.0)	75 (42.6)	360	

^1^ 162 missing samples, ^2^ 160 missing samples, ^3^ 160 missing samples, ^4^ 160 missing samples, ^5^ 161 missing samples, ^6^ 160 missing samples, ^7^ 160 missing samples, ^8^ 160 missing samples.

## Data Availability

All data generated or analyzed during this study are included in this published article.
